# Radiographic and histological evaluation of bone formation induced by rhBMP-2-incorporated biomimetic calcium phosphate material in clinical alveolar sockets preservation

**DOI:** 10.1186/s40729-023-00491-1

**Published:** 2023-10-16

**Authors:** Yuanyuan Sun, Chunfeng Xu, Mingjie Wang, Lingfei Wei, Herman Pieterse, Yiqun Wu, Yuelian Liu

**Affiliations:** 1 Department of Oral Cell Biology, Academic Center for Dentistry Amsterdam (ACTA), Vrije Universiteit Amsterdam and University of Amsterdam, Gustav Mahlerlaan 3004, 1081 LA Amsterdam, The Netherlands; 2 Department of Second Dental Center, Shanghai Ninth People’s Hospital, School of Medicine, College of Stomatology, Shanghai Jiao Tong University; National Center for Stomatology, National Clinical Research Center for Oral Diseases, Shanghai Key Laboratory of Stomatology, Shanghai, China; 3 Department of Oral Implantology, Yantai Stomatological Hospital, Binzhou Medical University, Yantai, China; 4 Profess Medical Consultancy BV, Heerhugowaard, The Netherlands

**Keywords:** Biomimetic, CBCT, rhBMP-2, Biopsy, Gray values, Bone regeneration, Histomorphometry

## Abstract

**Purpose:**

We assessed the efficiency of low-dose recombinant human bone morphogenetic protein-2 (rhBMP-2) incorporated biomimetic calcium phosphate on β-tricalcium phosphate (β-TCP) (rhBMP-2/BioCaP/β-TCP) on bone formation in a model of socket preservation using cone beam computed tomography (CBCT) scanning and histological examination.

**Methods:**

Forty patients undergoing minimally invasive single-root tooth extraction for dental implantation were randomized to three groups according to the material used for socket preservation: filling with rhBMP-2/BioCaP/β-TCP, β-TCP, or natural healing (kept unfilled) (controls). The alveolar sockets (including the control group) were covered by two-layer collagen membranes and sutured. Two CBCT scans were taken, one immediately after socket preservation procedure (baseline) and another 6 weeks later. Gray values (GVs) obtained from CBCT were recorded. During insertion of the dental implant, biopsies were taken and analyzed histologically for new bone formation, residual material, and unmineralized bone tissue at the core of the biopsy.

**Results:**

The mean (± standard deviation) changes of GVs of the CBCT scans at the central area of filled materials were as follows: 373.19 ± 157.16 in the rhBMP-2/BioCaP/β-TCP group, 112.26 ± 197.25 in the β-TCP group, and -257 ± 273.51 in the control group. The decrease of GVs in the rhBMP-2/BioCaP/β-TCP group as compared with the β-TCP group was statistically significant (*P* < 0.001). Differences in new bone formation (*P* = 0.006) were also found: 21,18% ± 7.62% in the rhBMP-2/BioCaP/β-TCP group, 13.44% ± 6.03% in the β-TCP group, and 9.49% ± 0.08% in controls. The residual material was10.04% ± 4.57% in the rhBMP-2/BioCaP/β-TCP group vs. 20.60% ± 9.54%) in the β-TCP group (*P* < 0.001). Differences in unmineralized bone tissue (*P* < 0.001) were also found (68.78% ± 7.67%, 65.96% ± 12.64%, and 90.38% ± 7.5% in the rhBMP-2/BioCaP/β-TC, β-TCP, and control groups, respectively).

**Conclusions:**

This study shows that rhBMP-2/BioCaP/β-TCP is a promising bone substitute with fast degradation and potent pro-osteogenic capacity that can be useful for socket preservation in implant dentistry.

*Trial registration*: ChiCTR, ChiCTR2000035263. Registered 5 August 2020, https://www.chictr.org.cn/ChiCTR2000035263.

**Graphical Abstract:**

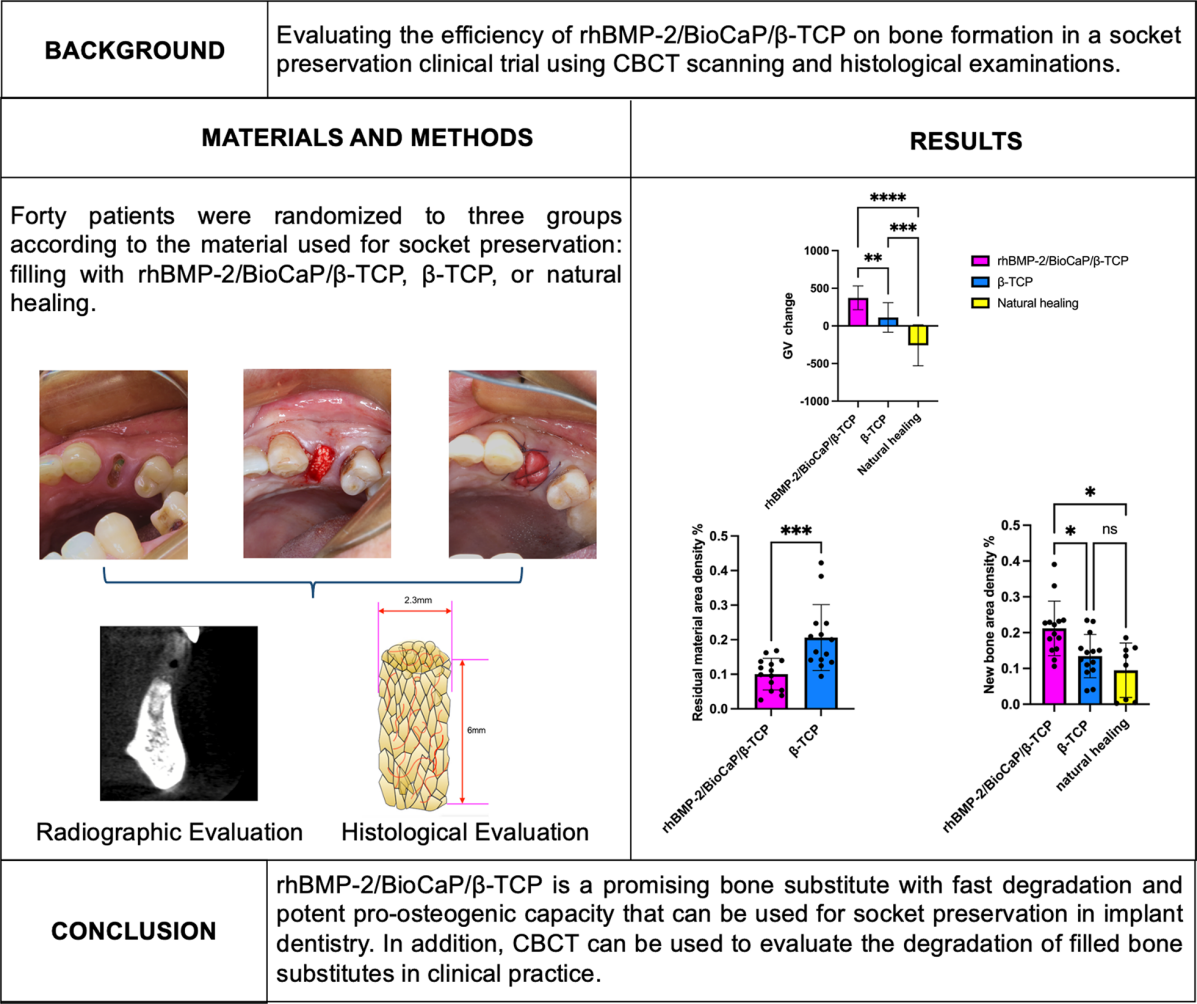

## Introduction

Sufficient alveolar bone is a prerequisite for successful placement of dental implants. However, atrophic maxilla or mandible is a common finding in clinical practice due to tooth loss, trauma, tumors, neoplasm resection, or bone metabolism diseases [1]. To provide adequate alveolar bone for dental implants, socket preservation has been widely used after tooth extraction, and many studies have shown that socket preservation procedures can reduce bone resorption and promote bone formation during the first 3 months after tooth extraction [2, 3].

Different materials, such as autografts, allografts, and xenografts have been used for filling the socket and retaining the alveolar bone volume [4]. Although autografts are considered the gold standard for bone regeneration [5], there are difficulties in obtaining sufficient bone from a single donor site, and the use of various sites involves additional surgery with longer operating and healing times, and increasing discomfort and morbidity [6]. Although using allografts is more feasible, spreading infection diseases is an inconvenience [7, 8] as well as the fact that, in some cultures, allografts and xenografts may be limited for religious reasons.

Synthesized bone substitutes are useful options as they can avoid these aforementioned shortcomings. Different synthesized bone substitute materials have been used in implant dentistry for socket preservation, such as hydroxyapatite (HA), tricalcium phosphate (TCP), biphasic calcium phosphate (BCP) or a combination of these materials. Calcium phosphate (CaP) bioceramics are mostly used as bone substitutes in clinical practice, and low dose of recombinant human bone morphogenetic protein-2 (rhBMP-2) (approved by the FDA) [9] added to β-TCP confers osteoinductivity and enhances the performance of this material in bone formation. Although the safety and efficacy of this rhBMP-2/BioCaP/β-TCP combination have been tested both in vitro and in vivo models [10, 11], data on its clinical performance are still limited.

Cone beam computed tomography (CBCT) is widely used in implant dentistry with grey values (GVs) scale to measure density, volume, and contour of bone [12]. However, GVs cannot accurately reflect the bone density when calcium phosphate-based preparations are used as filling materials. In these cases, bone density should be evaluated by histomorphometric techniques.

Therefore, the objective of this study was to determine the efficiency of rhBMP-2/BioCaP/β-TCP in socket preparation using CBCT studies and histological examination of biopsies for assessing bone formation.

## Methods

### Patient selection and study design

The study was approved by the Clinical Research Ethics Committees of the Academic Center for Dentistry of Amsterdam (code ACTA 202061), Vrije Universiteit Amsterdam, The Netherlands, and Shanghai Ninth People’s Hospital, Shanghai Jiao Tong University, School of Medicine (code SH9H-2019-T231-4), China. This trial was conducted following the international standard for clinical investigations with medical devices (ISO 14155:2020). Written informed consent was obtained from all participants.

**Fig. 1:**
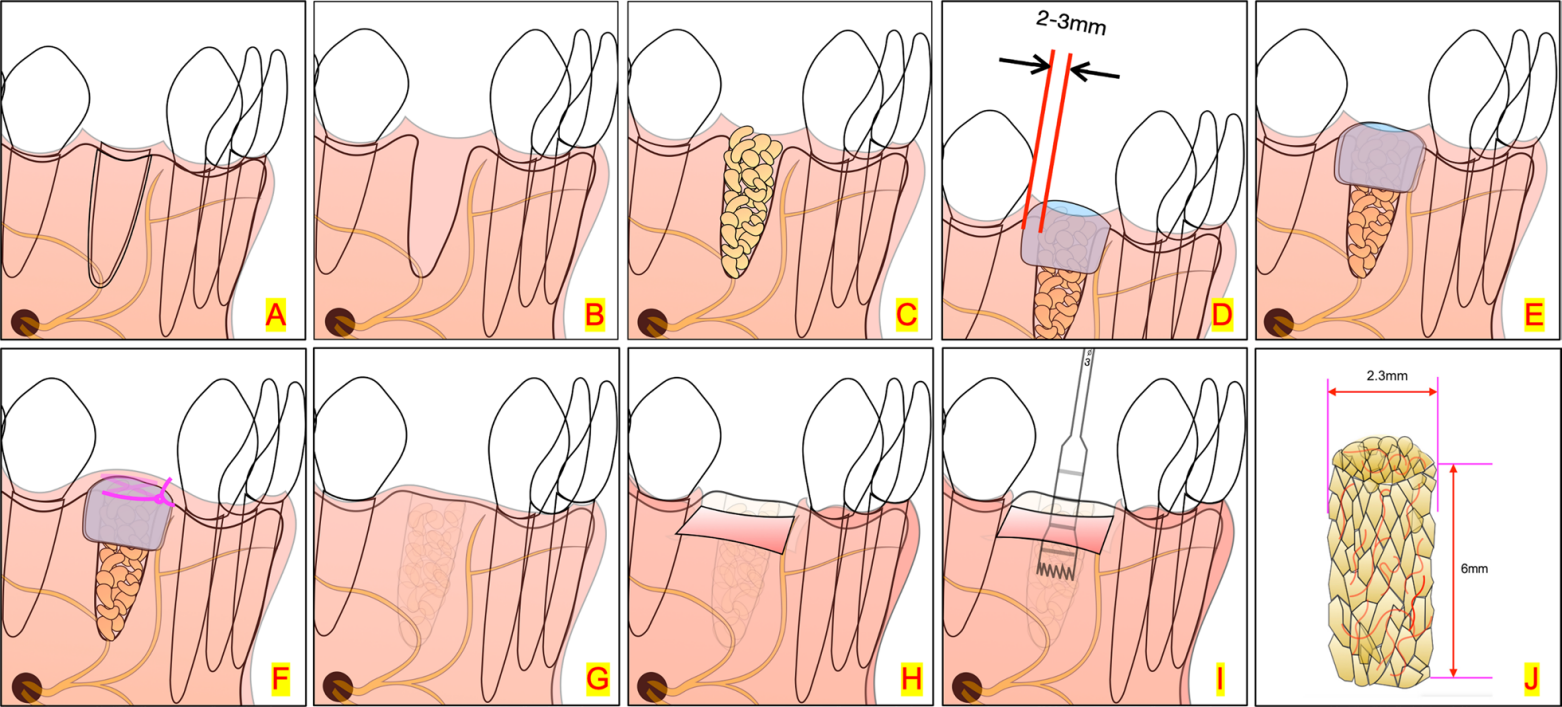
Schematic of clinical trial procedure. **A** The patients were randomly divided into rhBMP-2/BioCaP/β-TCP, β-TCP, and natural healing groups; **B** tooth extraction, **C** the tooth socket filled with either rhBMP-2/BioCaP/β-TCP or β-TCP in relative groups, **D** the first layer of collagen membrane covered the alveolar sockets and 2-3 mm over the socket edge), **E** the second collagen membranes, **F** sutured, **G** 2 weeks later, took out the suture, **H** after 6 weeks, soft tissue flap releasing, **I** a trephine drill (outer diameter 3 mm, inter 2.3 mm diameter) is used to obtain the biopsy, **J** collected biopsy (2.3 mm in diameter × 6 mm in height)

**Fig. 2:**
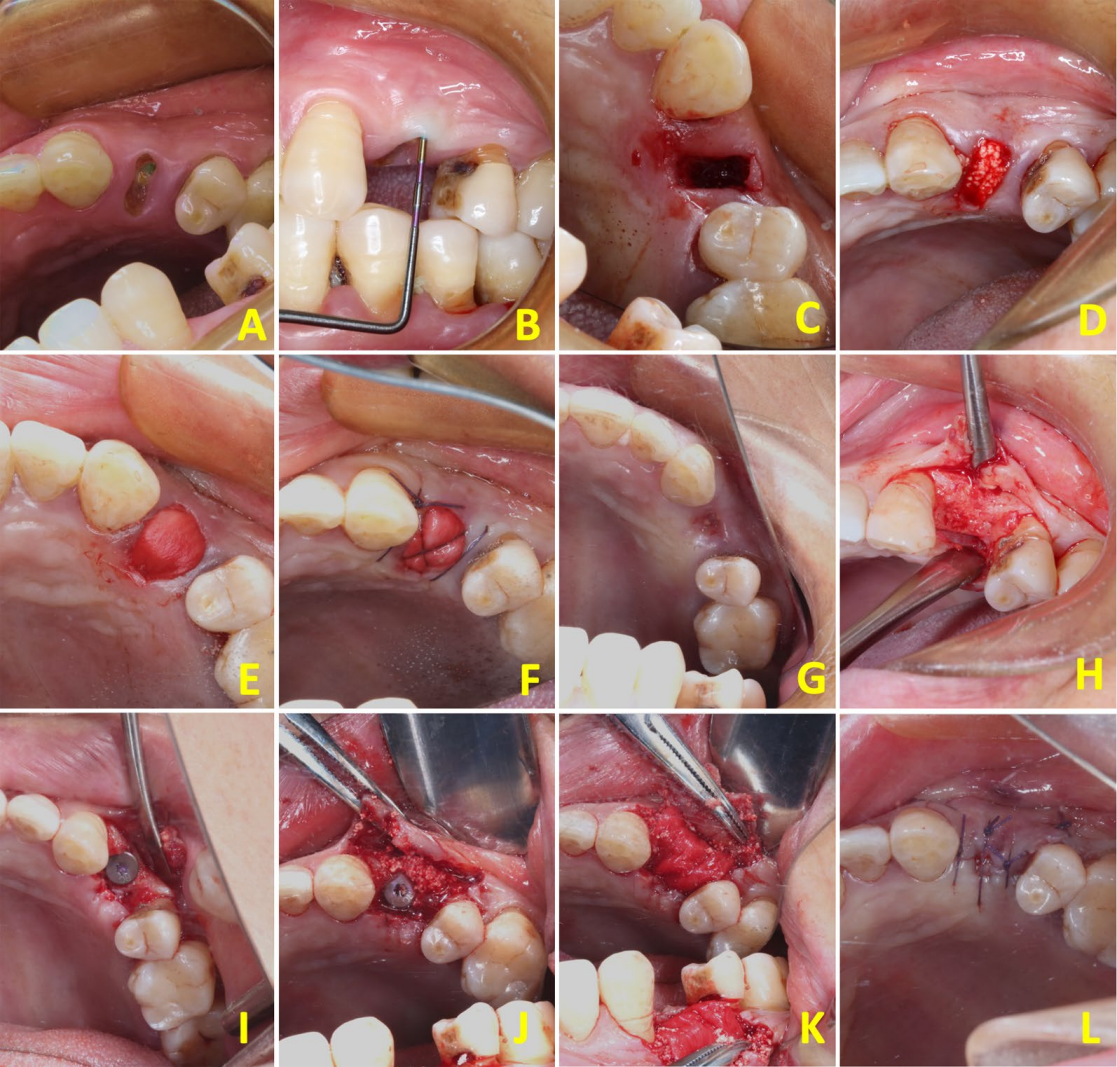
Intra-oral photographs of socket preservation and dental implant surgery. **A** The upper left first premolar needed to be removed. **B** The gingival biotype was a thick tissue biotype. **C** Tooth extraction. **D** The tooth socket was filled with rhBMP-2/BioCaP/β-TCP. **E** Two layers of collagen membrane covered the alveolar socket. **F** Sutured. **G** 6 weeks later. **H** Soft tissue flap releasing. **I** Obtained the biopsy and inserted an implant. **J**, **K** Guided bone regeneration (GBR) for the horizontal bone gain. **L** Sutured

A total of 40 patients were recruited in this study (15 in rhBMP-2/BioCaP/β-TCP group, 15 in β-TCP group, and 10 in the natural healing group). All patients met the selection criteria shown in Tables 1 and 2. The patients were randomly divided into the following three groups: rhBMP-2/BioCaP/β-TCP, β-TCP, and natural healing (kept unfilled) (controls). After tooth extraction, the socket was filled with either rhBMP-2/BioCaP/β-TCP (particle size 0.25–1 mm, made by Shanghai Rebone Biomaterials Co., Ltd.) or β-TCP (particle size 0.25–1 mm) β-TCP group, while the natural healing group was not filled with any CaP material. Two layers of collagen membrane (Geistlich Bio-Gide^®^ bilayer collagen membrane, 25 × 25 mm) were used to cover the filling materials in the alveolar sockets of all patients. The soft tissue was sutured. Immediately after surgery, the first CBCT scan was taken and the GVs were recorded. Six weeks after the procedure, a second CBCT scan was obtained before placement of the implant. In addition, a biopsy of 2.3 mm in diameter and 6 mm height was taken using a trephine drill (3 and 2 mm, outer and inner diameters, respectively) at the same point of the implant insertion (Figs. 1, 2). The operation was completed when the implant was inserted and the sutures were removed after 2 weeks.

**Table 1:** Inclusion criteria

• Patients over the age of 18 years • The study includes patients who have undergone the extraction of a single-root tooth based on appropriate indications for tooth extraction • Patients whose tooth extraction site has been classified as either EDS-1 or EDS-2 according to the classification system developed by N. Caplanis et al. [27] • After the tooth extraction, implant surgery will be the follow-up treatment for these patients • Before participating in the trial, all subjects provided their voluntary and informed consent to participate in the study

**Table 2:** Exclusion criteria

• Patients with conditions considered absolute contraindications for oral implant treatment based on the criteria established by Debby Hwang et al. [28] • Patients with uncontrolled local inflammation in the extracted or adjacent teeth, including uncontrolled periodontitis (periodontal probing depth > 4 mm) and acute periapical periodontitis • Patients with uncontrolled diabetes, where fasting blood glucose is still ≥ 8.8 mmol/L despite drug use • Patients with severe uncontrolled hypertension (blood pressure > 180/100 mmHg) • Obese patients with a body mass index (BMI) > 28 kg/m^2^ • Patients who have continuously used antibiotics or chronic anti-inflammatory therapy (≥ three times per week) within the first four weeks after surgery • Patients who smoke or use tobacco equivalent/chewing tobacco more than ten cigarettes daily • Patients who have an allergy to the investigational product • Female patients who are pregnant or nursing or refuse to take any contraception • Patients whose compliance could be improved by the investigator • Patients who have participated in or are participating in clinical trials of other medical devices or drugs within the 30 days before Day 0

### Radiographic measurement

**Fig. 3:**
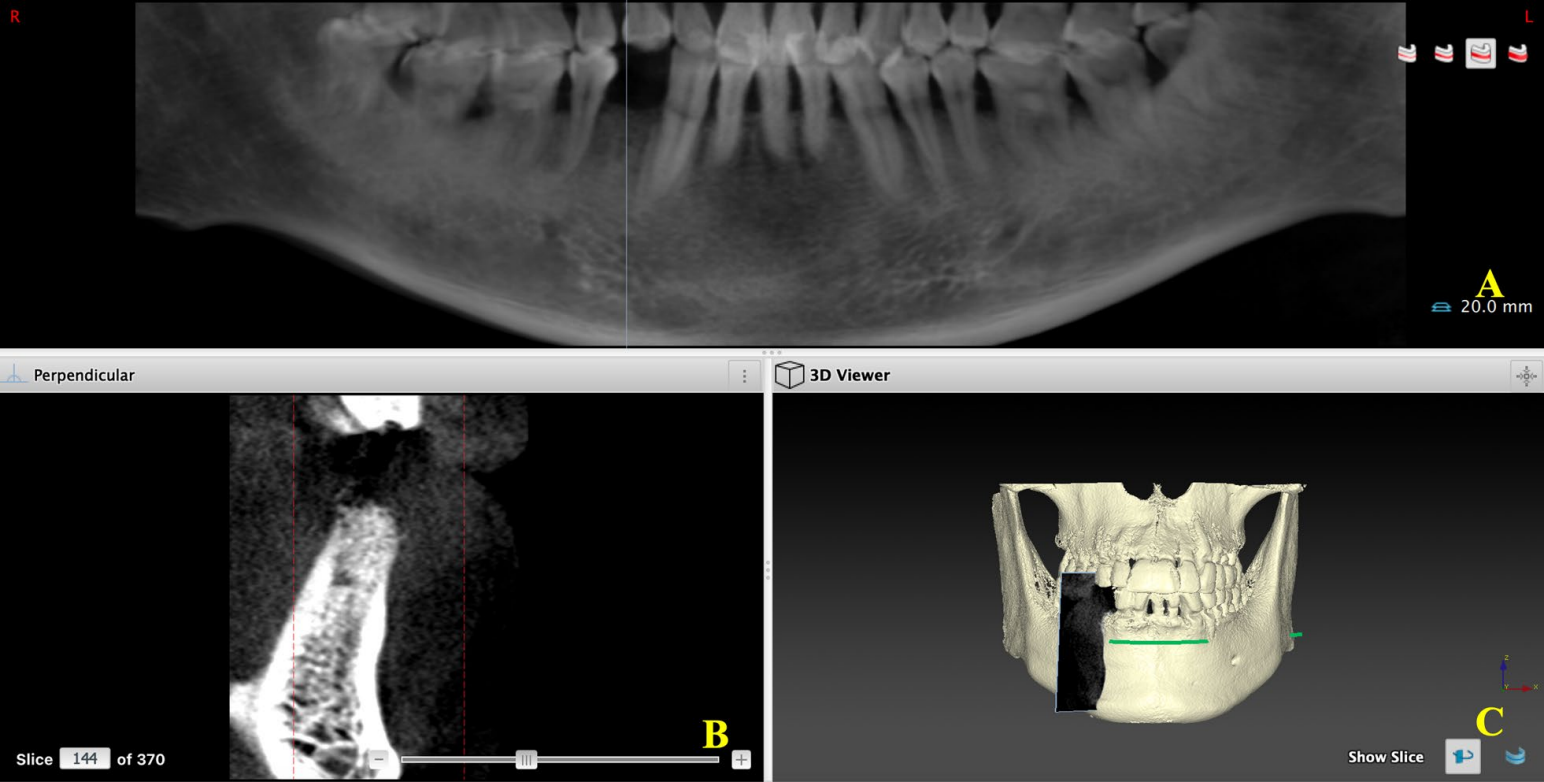
Data import. **A** Panoramic tomography. **B** Buccal-lingual section of the surgery area. **C** 3D model

**Fig. 4:**
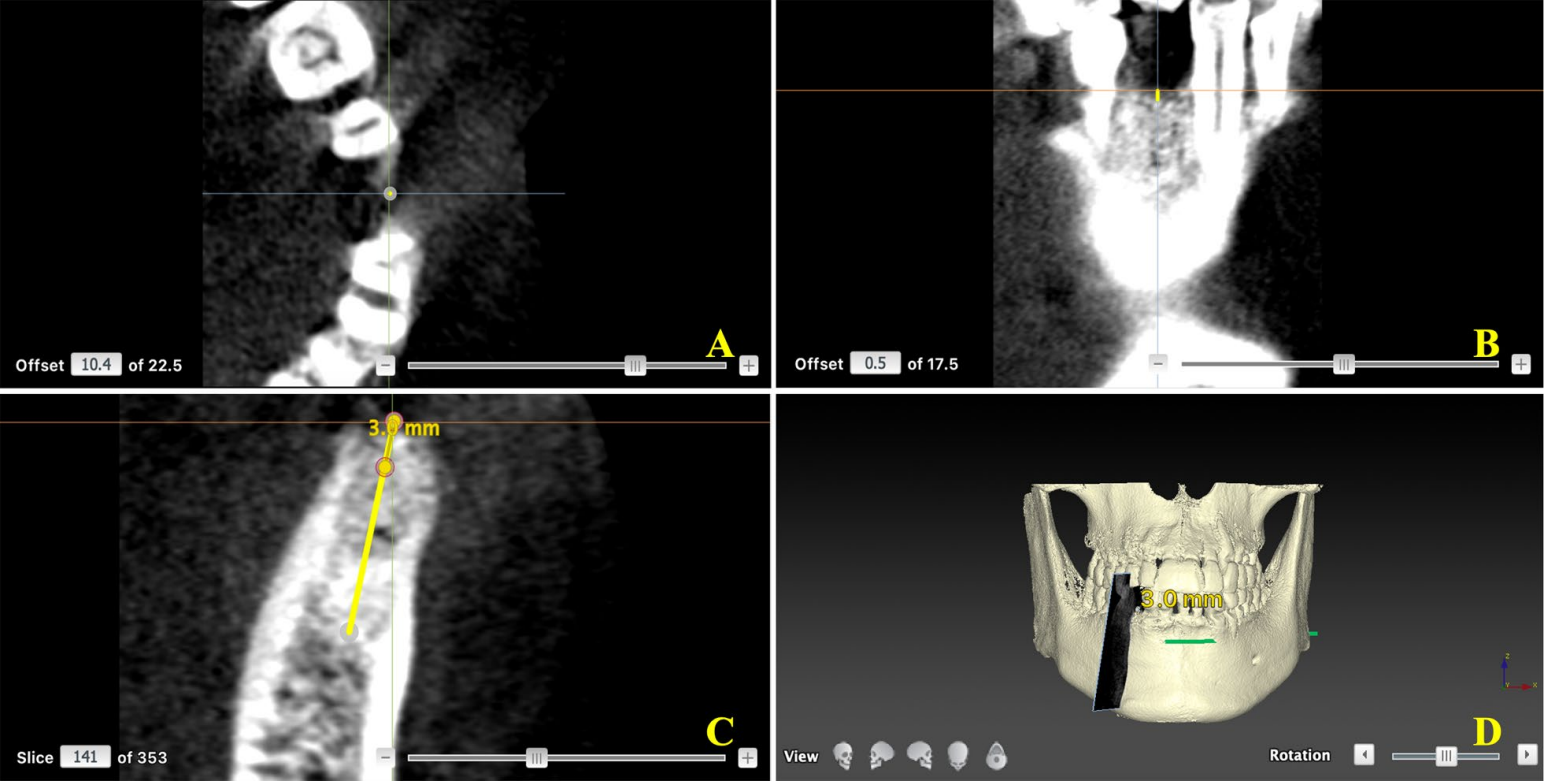
The measurement point of GV. **A** Look for the central point of the pulp cavity at the enamel-cementum junction of the mesial and distal adjacent teeth. The connection of the two points helps determine the coronal plane. **B** The horizontal reference line was taken through the highest alveolar ridge of extraction fossae (crest of the alveolar ridge). **C** The buccal-lingual section followed the center line of the root of the tooth. Then the tooth-long axis was followed to find the 3 mm point (GV measurement point) from the baseline and measured the GV based on the software function. **D** 3D view

**Fig. 5:**
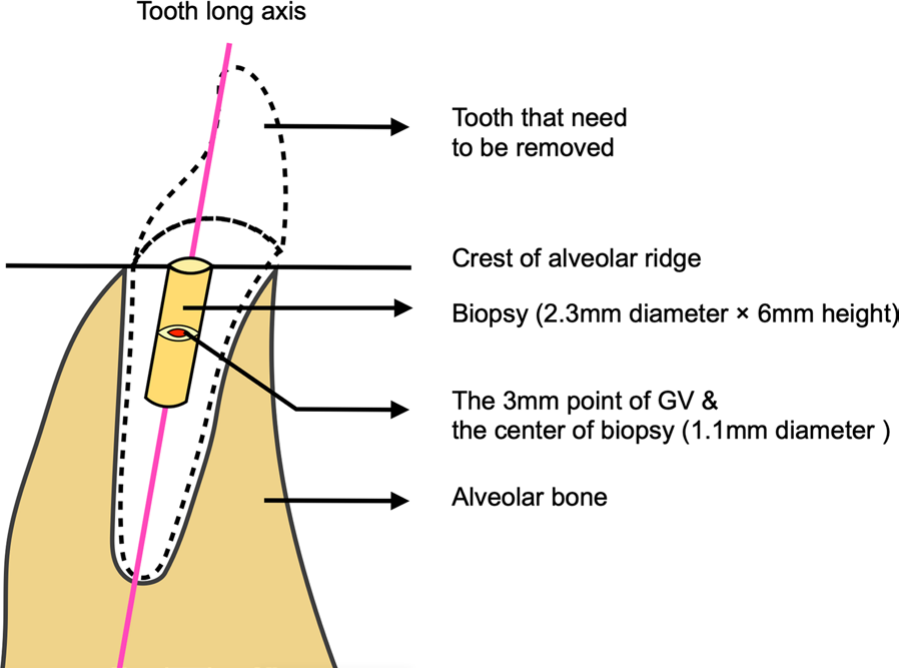
Schematic diagram of the identification of measurement point for GV. The GV measurement baseline is on the alveolar ridge’s crest. Following the long axis of the tooth, the red dot is a point for measuring bone density underneath 3 mm of baseline. The center part of the biopsy is the same as the GV-measured point of the CBCT image

The CBCT images were collected using the Planmeca 3D Imaging System (field-of-view of 8 cm (D) × 8 cm (H), resolution 0.16 mm, Planmeca, Finland). After being exported as digital imaging and communications in medicine (DICOM) files, the data were analyzed using planning software (Nobel Clinician, Nobel Biocare, Sweden) for GV measurement (Fig. 3). Three-dimensional images focused on the socket site to identify the central point of the pulp cavity at the enamel–cementum junction of the mesial and distal adjacent teeth were obtained. The connection of the two points helps to determine the coronal plane (Fig. 4A). The horizontal reference line was taken through the highest alveolar ridge of extraction fossae (crest of the alveolar ridge) (Fig. 4B). The buccal-lingual section followed the center line of the root of the tooth. Then the tooth-long axis was followed to identify the 3 mm point (GV measurement point) from the baseline, and the GV was measured based on the software function (Fig. 4C). A 3D model showed the 3D information (Fig. 4D), and the schematic diagram of the GV point is shown in Fig. 5. All measurements were performed by three independent investigators, and the GV change was calculated as the difference between baseline (GV1) and final GV obtained at 6 weeks after material filling (GV change = GV1 – GV2).

### Histomorphological examination

Tissue biopsies were immersed in 10% neutral formalin solution for 24 h with the trephines.

**Fig. 6:**
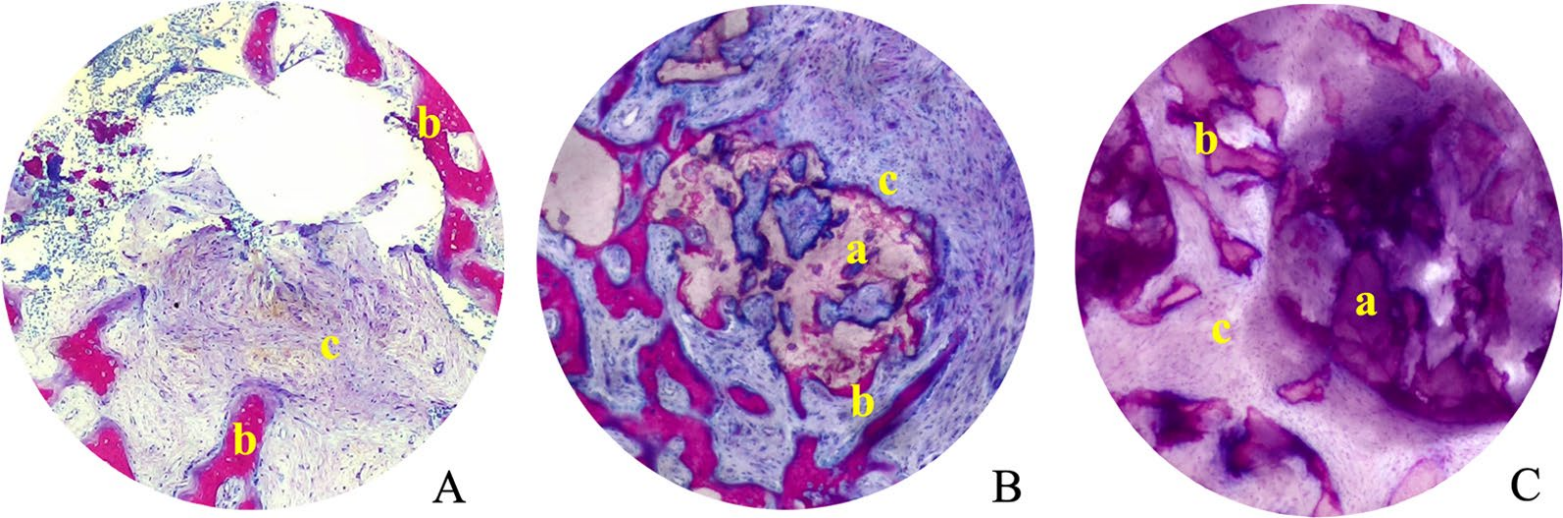
Light micrographs of biopsy slice (diameter 1.1 mm) in the natural healing group (**A**), rhBMP-2/BioCaP/β-TCPgroup (**B**), and β-TCP group (**C**) 6 weeks after implantation. Stained with McNeil’s Tetra chrome basic fuchsine and toluidine blue O. **a** Residual material, **b** new bone, **c** unmineralized tissue

Thereafter, samples were dehydrated with alcohol gradients after flushing, and embedded with polymethyl methacrylate. The samples were sectioned opposite to the long axis of the biopsy, and five tissue sections of 600 µm thicknesses (1 mm spacing) were collected for each piece, polished to a final thickness of 50–100 µm, and stained with McNeal’s tetrachrome staining. The Image Pro Plus program (version 6.0, Media Cybernetics) was used to calculate the area of new bone, the residual material, and the unmineralized tissue at the center slice of the biopsy (Fig. 6) The center part of the biopsy as the same as the GV-measured point of the CBCT image area was measured. Measurements were performed by three independent pathologists, and the mean value of these measurements was considered.

### Statistical analysis

Categorical data are expressed as frequencies and percentages, and continuous data as mean (± standard deviation, SD) or median and interquartile range (IQR) (25th–75th percentile). Analysis was performed in the per-protocol (PP) dataset, that is, all patients who were randomized, received the intervention, and completed the study procedures. A single-factor analysis of variance (ANOVA) was used to compare CVs changes among the three study groups. The Mann–Whitney *U* test was used to compare the area of residual material between rhBMP-2/BioCaP/β-TCP and β-TCP groups, and the Kruskal–Wallis test for the comparison of the new bone area and unmineralized tissue area among the three study groups. Interrater reliability was assessed with the Cronbach’s alpha (α) coefficient. Statistical significance was set at *P* ≤ 0.05. The IBM Statistical Package for the Social Sciences software (SPSS) (version 23.0) was used for the analysis of data.

## Results

Of the 40 patients recruited for the study, 4 (10%) were excluded due to protocol violations (prohibited medication history, significantly exceeding the follow-up time-point limit, and severely defective bone), 2 of them from the natural healing group, and 1 patient each from the rhBMP-2/BioCaP/β-TCP and β-TCP groups, respectively. Therefore, the study population included 14 patients in the rhBMP-2/BioCaP/β-TCP group, 14 in the β-TCP, and 8 in the control group.

### GV changes on CBCT images

In the first CBCT scan (baseline), there were no statistically significant differences in GVs between the rhBMP-2/BioCaP/β-TCP and β-TCP groups (Table 3). After 6 weeks of socket preparation, the GV change at the 3 mm point below the socket ridge showed significant statistical differences among the three groups (Table 4), and GV changes in the rhBMP-2/BioCaP/β-TCP group were significantly greater than in the β-TCP group (373.19 ± 157.16 vs.112.26 ± 197.25). The median GV (min, max) of the rhBMP-2/BioCaP/β-TCP group was 386.67 (157.33, 642.67) as compared with 67.67 (− 198, 443) in the β-TCP group (Table 5).

**Table 3:** Initial GV in β-TCP and rhBMP-2/BioCaP/β-TCP group

GV	β-TCP	rhBMP-2/BioCaP/β-TCP	Statistical method	Statistic	*P* value
*N* (missing)	14(0)	14(0)	*t*-test	*t* = 0.091	= 0.928
Mean ± SD	835.48 ± 203.05	841.19 ± 118.72			
Median	824.50	855.00			
Min, Max	520.33,1160.67	599.33,1026.67

**Table 4:** Comparison of CBCT data of the GV change in three groups

GV change	Natural healing	β-TCP	rhBMP-2/BioCaP/β-TCP	Statistical method	Statistic	*P* value
*N* (missing)	8	14(0)	14(0)	ANOVA	*F* = 24.83	< 0.001
Mean ± SD	− 257 ± 273.51	112.26 ± 197.25	373.19 ± 157.16			
Median	− 251.67	67.67	386.67			
Min, Max	− 594,61.33	− 198,443	157.33,642.67

**Table 5:** Comparison of CBCT data of the GV change in β-TCP group and RhBMP-2/BioCaP/β-TCPgroup

GV change	β-TCP	rhBMP-2/BioCaP/β-TCP	Statistical method	Statistic	*P* value
*N* (missing)	14(0)	14(0)	*t*-test	*t* = 3.871	< 0.001
Mean ± SD	112.26 ± 197.25	373.19 ± 157.16			
Median	67.67	386.67			
Min, Max	− 198,443	157.33,642.67			

### Histomorphological results

The percentage of new bone area at the 3 mm point was statistically significant different in the three study groups, with higher values in the rhBMP-2/BioCaP/β-TCP group (21.18% ± 7.62% in the rhBMP-2/BioCaP/β-TCP group, 13.44% ± 6.03% in the β-TCP group, and 9.49% ± 0.08% in controls). Also, the median (min, max) values were 20.93% (10.62%, 39.08%) in the rhBMP-2/BioCaP/β-TCP group, 13.48% (3.78%, 23.42%) in the β-TCP group, and 12.21% (0.28%, 18.58%) in the controls (Table 6). A comparison of the percentages of new bona areas showed statistically significant differences between the rhBMP-2/BioCaP/β-TCP group and the remaining two groups, but significant differences between the β-TCP and control groups were not found (Table 7).

**Table 6:** Comparison of histological data of the biopsies-PPS in three groups

	Natural healing	β-TCP	rhBMP-2/BioCaP/β-TCP	Statistical method	Statistic	*P* value
Residual material area %						
*N* (missing)		14(0)	14(0)	Mann–Whitney Test	*Z* = 3.354	< 0.001
Mean ± SD		20.60 ± 9.54	10.04 ± 4.57			
Median		18.24	10.47			
Q1, Q3		13.94,24.72	5.31,13.76			
Min, Max		9.38,42.22	2.58,16.80			
New bone area %						
*N* (missing)	8(0)	14(0)	14(0)	Kruskal–Wallis Test	*H* = 10.28	= 0.006
Mean ± SD	9.49 ± 0.08	13.44 ± 6.03	21.18 ± 7.62			
Median	12.21	13.48	20.93			
Q1, Q3	0.67, 15.60	9.41, 16.70	15.34, 23.25			
Min, Max	0.28, 18.58	3.78, 23.42	10.62, 39.08			
Unmineralized tissue area %						
*N* (missing)	8(0)	14(0)	14(0)	Kruskal–Wallis Test	*H* = 17.88	< 0.001
Mean ± SD	90.38 ± 7.50	65.96 ± 12.64	68.78 ± 7.67			
Median	88.00	67.43	70.44			
Q1, Q3	84.25, 99.00	62.84, 75.16	63.27, 75.49			
Min, Max	81,99	34.64, 81.69	51.35, 77.76

**Table 7:** Pairwise comparison of histological data of the new bone area %

Sample 1–Sample 2	Test statistic	Std. error	Std. test statistic	Sig	Adj. Sig.
3 vs 2	3.55	4.67	0.76	0.447	1.00
3 vs 1	13.48	4.67	2.88	0.004	0.012
2 vs 1	9.92	3.98	2.49	0.013	0.038

1: rhBMP-2/BioCaP/β-TCP group; 2: β-TCP group; 3: natural healing group

In the rhBMP-2/BioCaP/β-TCP group, the percentage of residual materials area was 10.04% ± 4.57%, the median (min, max) was 10.47% (2.58%, 16.80%), which was significantly lower than that observed in the β-TCP group, 20.60% ± 9.54%, median (min, max) was 18.24% (9.38%, 42.22%) (Table 6).

There were statistically significant differences in the percentage of unmineralized tissue area among the three groups (the rhBMP-2/BioCaP/β-TCP group: 68.78% ± 7.67%; the β-TCP group: 65.96% ± 12.64%; and the natural healing group: 90.38% ± 7.5%) (*P* < 0.001) (Table 6). Comparing within groups, the percentage of unmineralized tissue area in both the rhBMP-2/BioCaP/β-TCP group and the β-TCP groups were significantly lower than in controls, but there was no statistically significant differences between the 2/BioCaP/β-TCP and the β-TCP groups (Table 8).

**Table 8:** Pairwise comparison of histological data of the unmineralized tissue area %

Sample 1–Sample 2	Test statistic	Std. error	Std. test statistic	Sig	Adj. Sig.
2 vs 1	0.786	3.981	0.197	0.844	1.00
2 vs 3	− 18.232	4.668	− 3.906	< 0.001	0.000
1 vs 3	− 17.446	4.668	− 3.737	< 0.001	0.001

1: rhBMP-2/BioCaP/β-TCP group; 2: β-TCP group; 3: natural healing group

## Discussion

This study used CBCT scanning and histological examination to evaluate degradation and new bone formation associated with the use of rhBMP-2/BioCaP/β-TCP and β-TCP as filling materials in socket preservation. CBCT scanning showed that there was a greater GV decrease in the rhBMP 2/BioCaP/β-TCP group than in the β-TCP group, which suggest a faster degradation rate of this material. In addition, less residual material and more new bone formation were identified in rhBMP-2/BioCaP/β-TCP group based on histomorphometric examination.

In both CBCT and biopsy tissue samples, the center of the filled area was selected to assess bone density, the volume of residual material, and new bone, as there were fewer interference factors (soft tissue and old bone tissue) around the target sites. Since the biopsies were 6 mm high, 3 mm under the reference of the alveolar ridge and alongside the tooth-long axis was included for GV change measurement, and CBCT results revealed that rhBMP-2/BioCaP/β-TCP group has more GV decrease in CBCT images, which indicated faster degradation than β-TCP group, and this assumption was confirmed in histomorphometric evaluations. Despite the CBCT results were consistent with those from histomorphometric assays. GV from CBCT alone cannot precisely reflect bone density, as GV quantifies the amount of X-ray attenuation of bone tissue and filled materials. Therefore, CBCT cannot independently determine the occurrence of new bone development.

The faster degradation of rhBMP-2/BioCaP/β-TCP may be attributed to more cell adhesion. In biomaterial-induced bone regeneration, biomaterials act as scaffolds where bone cells and osteoclasts can adhere and grow [13, 14]. The osteoclast-induced degradation of biomaterials has been extensively reported [15]. Although pore size, porosity, and the roughness of rhBMP-2/BioCaP/β-TCP may benefit cell adhesion including osteoclasts adhesion, further studies are needed to assess these characteristics of rhBMP-2/BioCaP/β-TCP.

Multislice computed tomography (MSCT) and micro-CT have been widely used to evaluate bone density, using the Hounsfield unit (HU) values, in orthopedics and laboratory studies [16]. However, they are not popular in dental clinical practice. Alternatively, CBCT is prevailing in evaluating the contour of alveolar bone before implant surgery, due to lower radiation, simpler design, and acceptable image resolution [17]. Some researchers compared HU from MSCT with GV from CBCT and concluded that the GV was more reliable for detecting and analyzing hypodense structures [18]. For bone evaluations, it was reported that high-resolution CBCT could be used for imaging quantitative bone morphometry assessment [19] and suggested there is a positive correlation between the GVs and the HUs values in quantifying bone tissue [20]. Similarly, it was reported that CBCT was as accurate and reliable as MSCT in predicting bone density and assessing changes in bone density around dental implants [21]. In 2013, radiographic bone density obtained from CBCT was compared with bone volumetric/total volume from micro-CT, and the result showed that it has a strong positive correlation with these two measurements. This study concluded that CBCT is a dependable tool for evaluating bone density preoperatively [22]. We here explored the potential application of CBCT in bone density evaluation in socket preservation. The results also implied CBCT is a potential tool to analyze the degradation of bone substitutes. However, the surgery of obtaining the biopsy was done by free hand, so the measurement points of the two methods cannot be guaranteed to coincide exactly. Next step, we will use the digital design and digital surgery guide technology to improve the consistency of the measurement positions of the two methods.

Alveolar ridge preservation can provide adequate alveolar bone for dental implants [23–26].

In this study, patients with extraction defect assessment (EDS) class 1 (EDS-1) or 2 (EDS-2), having less bone loss than those with EDS-3 or EDS-4, were included after tooth extraction, and they were in a better condition for bone healing. The rhBMP-2/BioCaP/β-TCP group was superior to β-TCP and natural healing (control) groups in bone formation. However, to assess the efficiency of rhBMP-2/BioCaP/β-TCP in adverse conditions for bone regeneration (e.g., EDS-3 and EDS-4), more studies are warranted.

## Conclusion

rhBMP-2/BioCaP/β-TCP is a promising bone substitute with fast degradation and potent pro-osteogenic capacity and can be used for socket preservation in implant dentistry. In addition, CBCT is a valuable technique to evaluate the degradation of filled bone substitutes in clinical practice.

## Data Availability

The datasets used and/or analysed during the current study are available from the corresponding author upon reasonable request.
